# DNA Nanotweezers with Hydrolytic Activity for Enzyme-Free and Sensitive Detection of Fusion Gene via Logic Operation

**DOI:** 10.1155/2018/4178045

**Published:** 2018-10-18

**Authors:** Yongjie Xu, Xiangrong Luo, Nana Geng, Mingsong Wu, Zhishun Lu

**Affiliations:** ^1^Department of Laboratory Medicine, Guizhou Provincial People's Hospital, College of Basic Medicine, Guizhou University, Guiyang 550002, Guizhou, China; ^2^Special Key Laboratory of Oral Diseases Research, Higher Education Institutions of Guizhou Province, Zunyi Medical University, Zunyi 563099, Guizhou, China

## Abstract

Gene fusion is a molecular event occurring in cellular proliferation and differentiation, and the occurrence of irregular fusion gene results in various malignant diseases. So, sensing fusion gene with high performance is an important task for integrating individual disease information. Here, we proposed a nonenzymatic and high-throughput fluorescent assay system for the detection of fusion gene by employing DNA nanotweezers with hydrolytic activity. This tweezer was assembled by three single-stranded DNAs and engineered with sensing elements and reporting subunits. In the absence of the fusion gene, the engineered tweezer remained opened and inactive which led to no signal output. However, the addition of fusion genes would cause structure alterations of the tweezer from open to close and further DNAzyme activation with the assembly of two reporting subunits. Then, the activated DNAzyme catalyzed fluorescence substrates for signal conversion. Taking BCR/ABL fusion gene as an example, the tweezer-based assay system showed not only excellent distinguishing capability towards different input targets but also high sensitivity with a detection limit of 5.29 pM. In addition to good detection performance, this system was simple and enzyme-free, offering a powerful nanometer tool as a smart nanodevice for sensing fusion detection.

## 1. Introduction

Gene fusions are a molecular event in cancer [1–3]. Many fusions resulting from chromosomal rearrangements are driver mutations in tumors and are currently used as biomarkers or drug targets. Examples include BCR/ABL, a target for Gleevec in chronic myeloid leukemia [4]; EML4-ALK, a target for crizotinib in lung cancer [5]; and PAX3-FOXO1, a biomarker for alveolar rhabdomyosarcoma [6]. Meanwhile, gene fusions are also a necessary molecular event in the defense of cancer and other diseases. Such T-cell receptor excision circles (TRECs) and K-deleting recombination excision circles (KRECs), as circularized DNA elements, are formed during the fusion process that creates T- and B-cell receptors [7, 8]. Their quantity in peripheral blood can be considered as an estimation of thymic and bone marrow output, which reflects individual immunity as hallmarks. Therefore, detecting fusion gene with high sensitivity and specificity is an urgent need for clinical diagnosis.

Conventional methods for detecting fusion gene include real-time quantitative reverse transcription PCR [9], flow cytometry [4], chromosome analysis [5], fluorescence in situ hybridization [10], and more. Such methods are still time-consuming and complicated in operation to some extent. To overcome these limitations, biosensing methods have attracted substantial research efforts, and numerous electrochemical, chemiluminescent, electrochemiluminescent, fluorescent, surface plasmon and resonance biosensing systems have been developed. These methods facilitate fusion gene analysis and improve analytical performance to some extent by adopting enzyme-assisted isothermal amplification and nanomaterials. However, native enzymes and artificial nanomaterials usually suffer from instability and high cost, which put constraints on their further application. In addition, fusion event occurred in cellular development, and proliferation is similar to the “AND” logic gate event in computer science which can be harnessed for intelligent and versatile detection. Unfortunately, this uniform trait has not been well taken into consideration in these biosensing strategies. Therefore, the exploration of a smart method that meets these challenges simultaneously remains a challenge.

DNA molecules are of great utility for this purpose because the combinatorial sequence space allows for an enormous diversity of signal carriers [11], and the predictability and specificity of Watson–Crick base pairing facilitate the design of gate architectures [12]. As a versatile construction material, DNA molecules indeed have been used for engineering molecular structures, engineering biological nanodevices [13, 14], and engineering various nanodevices, including “tweezers” [15, 16], “walkers” [17, 18], “stepper” [19], and engineering more [20–22] mechanical functions through encoding information in the base sequence of DNA. These assemblies also have the ability to attain cascade amplification and logic gate operation upon including catalytic [23–25] and logical control elements [26–28] and circuits [29–32]. Besides, owing to their properties of high biocompatibility, outstanding stability, low cost, and easily custom synthesis, DNA-based assemblies have the potential to be powerful tools for biosensing and bioanalysis. It is noted that DNA tweezers are molecular devices that can sense, hold, and release target DNA upon specific interaction. Since the first demonstration of a DNA-fueled molecular tweezer by Yurke et al [33] based on the strand displacement mechanism, several DNA molecular tweezers operating on similar fashion have been reported. These DNA tweezers include the adenosine monophosphate and adenosine deaminase-triggered aptamer tweezers [34], the pH-programmable tweezers reversibly switched by pH stimuli [35], and the photo-responsive DNA tweezers operated by invertible photoswitching [36]. The operations of these tweezers, however, require either the involvement of enzymes which may be subject to thermodynamic limitations, or rigid pH control of the system which suffers from tedious preparation processes, or the use of toxic azobenzene moieties. The development of simple and cost-effective DNA tweezers with new functionality will therefore facilitate the construction of different molecular machines for various applications.

In the present study, we report a new type of smart DNA nanotweezer with catalytic function for specific recognition of BCR/ABL fusion gene and then outputting an amplified signal. The DNA nanotweezer, self-assembled from three single-stranded DNAs, is tailored with recognition elements and catalytic subunits which show promising switches for molecular computation and signal amplification [37]. Recognition elements respond to fusion gene logically, whereas the responses regulate the “open” and “closed” states of the tweeters and further regulate the “inactive” and “active” states of the DNAzyme. The activated DNAzymes successively cleave the fluorescence substrates, thus enabling the intelligent and sensitive detection of fusion events without the attending of any native enzyme.

## 2. Experimental Section

### 2.1. Reagents and Materials

The oligonucleotides used in the experiments were supplied by Shanghai Sangon Co., Ltd. (Shanghai, China). Fluorescence substrates of MNAzyme were purified with high-performance liquid chromatography (HPLC), while other strands were purified by polyacrylamide gel electrophoresis (PAGE). The base sequences of these oligonucleotides are given in Table S1. All oligonucleotides were prepared by resuspending the lyophilized oligonucleotides in DEPC-treated water at a nominal 10 *μ*M concentration and stored at −20°C until use. All other reagents were of analytical grade, and ultrapure water (≥18 MΩcm, Milli-Q, Millipore) was used in all experiments.

### 2.2. Apparatus

The gel electrophoresis was carried out in a flat-bed electrophoresis system on the PowerPac™ Basic electrophoresis analyzer (Bio-Rad, USA). The gel was imaged on the Bio-Rad ChemiDoc™ MP imaging system under ultraviolet light (Bio-Rad, USA), and florescence images were collected by the Bio-Rad ChemiDoc™ MP imaging system at an excitation wavelength of Cy5 (650 nm).

### 2.3. Agarose Gel Electrophoresis

The assembly process of the DNA nanotweezer and its response to target input were tested by agarose gel electrophoresis which was conducted in 1 × TBE buffer (90 mM Tris-HCl, 90 mM boric acid, 2 mM EDTA, and pH 7.9) at 150 V for 30 min. Then the gel was imaged with the ChemiDoc™ MP imaging system.

### 2.4. Nanotweezer Preparation

Prior to detection, the open nanotweezer was prepared by mixing stoichiometric quantities of stock solutions of strands A, B, and C to a final concentration of 1 *μ*M in a microtube. After that, the mixture was left for 30 min at room temperature to allow the reaction to reach near-completion. The assembled nanotweezer was then used for the sequent experiment.

### 2.5. Determination of Target Fusion Gene

For target fusion gene measurement, 5 *μ*L of 1 *μ*M DNA nanotweezer and 2 *μ*L of 10 *μ*M fluorescence substrates (Strand F, Cy5-GTTTCCTCguCCCTGG-BHQ1) were firstly transferred into wells of the microplate followed by adding different concentrations of BCR-ABL fusion gene. The mixed solution was carried out in 50 *μ*L of 1 × GeneAmp® PCR Buffer II with Mg^2+^ ion (10 mM Tris-HCl, 50 mM KCl, and 50 mM MgCl_2_; pH 8.3) and incubated at 40°C for 50 min. Then the resultant solutions were subjected to the Bio-Rad ChemiDoc™ MP imaging system at the excitation wavelength of 650 nm, and the fluorescence images at the emission wavelength of 670 nm were collected from the integration of fluorescence intensities for 10 s. Finally, the captured images were analyzed with the software of quantity one (Bio-Rad) to get the quantitative intensities. All the measurements were run in triplicates, and the assay system without target fusion gene was used as the blank control. All measurements were performed at room temperature.

## 3. Results and Discussion

### 3.1. Design of the Smart Nanodevice

**Scheme 1:**
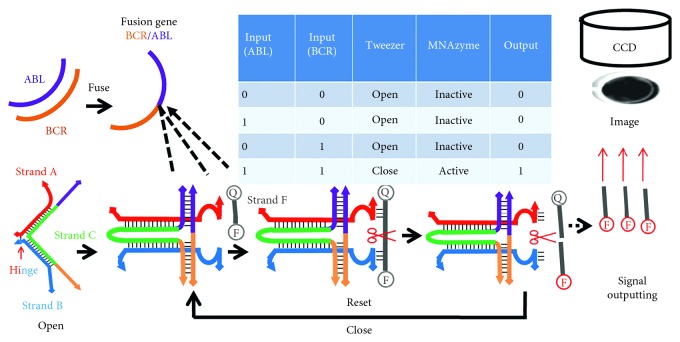
Schematic illustration of the fluorescence imaging method based on functional DNA nanotweezer regulated by fusion gene.

The design of the smart nanotweezer for nonenzymatic and sensitive detection of fusion gene is illustrated in Scheme 1. The nanotweezer is assembled from three single-stranded strains, termed as strands A, B, and C, respectively. Strand C consists of two 18-base sequences which hybridize with complementary sequences at the ends of strands A and B to form two stiff arms; the hinge is formed from a four-base single-stranded region of C between the regions hybridized to strands A and B. The free ends of strand C are loaded with sequences complementary to BCR and ABL sequences, respectively, served as reorganization elements for fusion gene. Meanwhile, strands A and B were designed with catalytic subunits which are at the distal terminus of the tweezers. In the absence of BCR gene and ABL gene (0, 0), the tweezers remain in the open state which is a catalytically inactive state so no signal output (0). Meanwhile, in the presence of BCR gene or ABL gene (0, 1 or 1, 0) separately, the tweezers remain in a catalytically inactive state and produce zero signal outputs (0). Rather, once BCR gene and ABL gene are linked together and form BCR/ABL fusion gene due to chromosomes rearrangement events (1, 1), the tweezers are closed since the binding between recognition elements and BCR/ABL fusion gene pulls the ends of the tweezers together. Thereafter, the stiff arms of the tweezers are held together by the closing strand, and catalytic activity is activated due to the proximity of the two DNAzymes subunits attached to a single strand of DNA, forming active DNAzyme structures. The DNAzymes cleave the fluorescent substrates and generate signal output (1). The alterations between active DNAzyme structures and a catalytically inactive nanostructure corresponding to molecular events easily attain logical operation, and the nanodevice smartly reflects the gene expression behavior of each individual cell.

### 3.2. Confirmation of Nanotweezers Abilities

**Figure 1:**
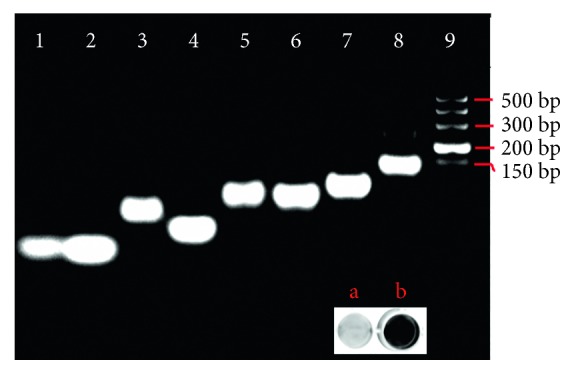
Characterization of the assembly process of DNA nanotweezer using agarose gel electrophoresis. Lanes 1, 2, 3, and 4 represents strand A, strand B, strand C, and BCR/ABL fusion gene, respectively, and lanes 5 and 6 are strand A + C and strand B + C, lane 7 represents opened nanotweezer assembled from strand A + B + C, and lane 8 represented closed nanotweezer after adding BCR/ABL fusion gene to the system in lane 7. Inset: the images a and b in lanes 7 and 8 represents the nanotweezer-based assay system without and with BCR/ABL fusion gene, respectively.

To confirm the construction of the functional nanotweezers, we used agarose gel electrophoresis to compare closed tweezers' (A, B, C) + target fusion gene with dimmers of (A, B, C), (A, C), and (B, C).  Figure 1 shows an image of electrophoresis bands. The bands in lanes 1, 2, 3, and 4 represent strand A, strand B, strand C, and BCR/ABL fusion gene, respectively, which are experimental controls. Lanes 5 and 6 correspond to incomplete structures of strand A + C intermediate and strand B + C intermediate. Lanes 7 contains open tweezers assembled by strands A, B, and C, while lane 8 contains closed nanotweezers corresponding to additions of the target fusion gene. Lane 9 stands for a 500 bp DNA ladder. In comparison with control bands, these bands from lane 5 to lane 8 show decreasing electrophoresis mobility with successive addition of different strands as nanotweezer components. The reason for the results is due to the stepwise assembly of nanotweezer components to form high-molecular-weight DNA hybrids. These results indicate the successful construction of the nanotweezers as sensing nanodevices and proper responses of the nanotweezers to target gene according to Scheme 1.

To validate the ability of nanotweezers acting as an “AND” logic operation device, BCR/ABL fusion gene as inputs were incubated with the DNA tweezers, and the fluorescence responses were recorded. The inset in Figure 1 depicts the signal responses. The control sample without the analyte or its analogues used for measuring the background fluorescence shows negligible signal (image a). However, the smart nanodevices gave a largely increased signal upon the addition of BCR/ABL fusion gene (image b). This is because the fusion process put the separated BCR gene and ABL gene near each other and further put the opened stiff tweezers with arms closed after interaction with the recognition elements. These results confirm that the nanotweezers pose the ability to sense fusion gene via switchable activity of different nanostructure states.

### 3.3. Optimization of Experimental Conditions

**Figure 2:**
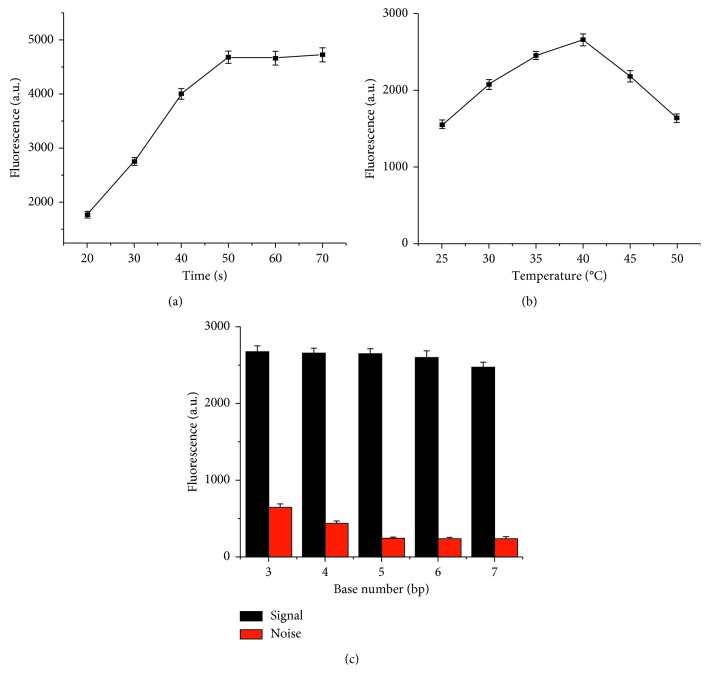
Optimization of experimental conditions. (a) Time-dependent fluorescence changes from 20, 30, 40, 50, 60, to 70 min. (b) DNAzyme cleavage activity on varied reaction temperature at 25, 30 35, 40, 45, and 50°C. (c) The effect of bases number on hinge region on signal to noise by setting at 3, 4, 5, 6, and 7 nucleotides.

To gain optimum assay performance, several important experimental parameters were optimized including incubation time, reaction temperature, and the number of bases on the hinge region. The time-dependent fluorescence changes were firstly measured through fusion gene detection experiments at 30, 40, 50, 60, and 70 min. As shown in Figure 2(a), along the prolonged time, the signal showed a gradual increase and tended to plateau at 50 min. So, the time period of 50 min was chosen for subsequent experiments. Since reaction temperature affects DNAzyme cleavage activity [38], we then tested the temperature effect on DNAzymes activity over a broad temperature range from 25, 30, 35, 40, to 45°C.  Figure 2(b) shows that the highest fluorescence intensity was obtained at 40°C which can be explained by the high DNAzyme activity when the temperature is most suitable for associating and disassociating between the DNAzyme substrate strands. Consequently, to avoid the unspecific close of the nanotweezers which would lead to a rise in the background signal, we designed a complementary sequence on the hinge which kept the tweezer fixed and opened by strands binding with each other. The number of sequence bases was optimized by measuring the signal responses of signal and noise when the bases are at 4, 5, 6, 7, and 8, respectively. As presented in Figure 2(c), the number of bases in the hinge showed an optimum at around 5 bases for the following assay.

### 3.4. Analytical Performance

**Figure 3:**
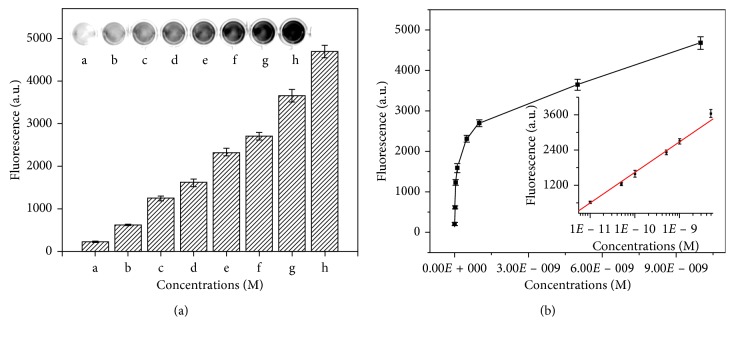
(a) Fluorescence images of BCR/ABL fusion gene at 0, 1 × 10^−11^, 5 × 10^−11^, 1 × 10^−10^, 5 × 10^−10^, 1 × 10^−9^, 5 × 10^−9^, and 1 × 10^−8^ M (image and bar a to h). (b) Dose-response curves for BCR/ABL fusion gene from 1 × 10^−11^ to 1 × 10^−8^ M. Inset: the corresponding calibration curve.

Under the optimized experimental conditions, the analytical performance of the catalytic sensing system was investigated. Both the concentrations of the nanotweezers and fluorescence substrates were fixed at 100 nM upon incubation for 50 min at 40°C. Then the fluorescence intensities were also measured in response to different concentrations of fusion gene from 0 to 10 nM. As shown in Figure 3(a), it can be observed clearly that the fluorescence signals rose with the increase of fusion gene mimics concentration. The fluorescence intensity was linearly dependent on the logistical value of fusion gene concentrations in the range of 0.01–10 nM, and there is a linear regression equation of *F* = 12141.35 + 1049.68 log_10_C, where *F* is the fluorescence signal and *X* is the concentration of fusion gene (*R* ^2^ = 0.9971). Based on the signal to noise ratio repeated three times, the limit of detection was estimated to be 5.29 pM. Then a comparison was conducted in PCR [39, 40] and PCR-free format [41, 42] that employed DNAzyme as molecular switches. Although PCR-based methods were more sensitive, this proposed strategy showed a low detection limit and a wide linear range which can be attributed to the logical signal response and catalytic MNAzyme-assisted signal amplification strategy.

**Figure 4:**
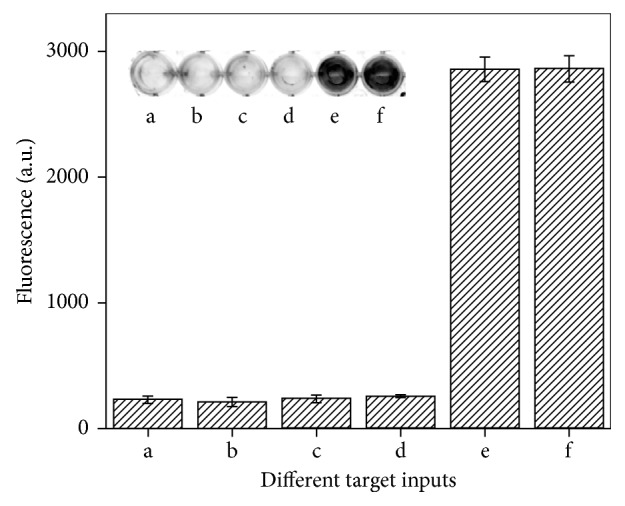
Fluorescence responses to different target inputs: random sequence (image and bar a), blank control (image and bar b), ABL gene (image and bar c), BCR gene (image and bar d), BCR/ABL fusion gene (image and bar e), and the mixture of ABL gene, BCR gene, and BCR/ABL fusion gene (image and bar f).

In addition to sensitivity, specificity of the assay system was also evaluated. Because in one individual cell, both BCR gene and ABL gene separately coexist even if no molecular event of chromosomes rearrangement occurs, the detection specificity was challenged by measuring the signal towards different targets as logic inputs, including a random DNA sequence, BCR gene, ABL gene, BCR/ABL fusion gene as well as the mixture of BCR gene, ABL gene and BCR/ABL fusion gene. As depicted in Figure 4, the smart nanodevice gives a different action towards different targets. The presence of a random DNA sequence and the absence of any target which were taken as inputs (0, 0) caused no signal output (images a and b). However, in the presence of BCR gene (1, 0) or ABL gene (0, 1), negligible signal changes in fluorescence were observed (0) as shown in images c and d, respectively. In these situations, binding of each existing gene with tweezers was taken as one separate logic input and thus lose the ability to pull the opened tweezers to be closed active DNAzymes structures. In contrast, the presence of BCR/ABL fusion gene (1, 1) that resulted from molecular fusion events led to significantly increased output signal (1) as shown in image e. These results validated the distinctive discrimination ability of nanotweezers acting as an “AND” logic operation device, demonstrating the good specificity of the smart nanosystem. This result is ascribed to the formation mechanism of the closed tweezers which rely on simultaneous recognition and binding with the fusion gene. Such an operating mechanism for the close of tweezers needs the fusion of two target inputs, which avoids the unspecial close induced by two separate signal inputs coexisting in one circumstance.

### 3.5. Recovery Test

To evaluate the accuracy and application potential of the proposed DNA nanodevice-based imaging system for fusion gene detection, recovery experiments were performed via spiking BCR/ABL fusion gene into100-fold diluted human serum samples. The assay solutions were incubated in a dark circumstance for 50 min at 40°C and then immediately imaged with the fluorescence imaging system with a cooled CCD camera. As shown in Table 1, fluorescence intensities resulted from six parallel experiments generated an almost identical value to the spiked standard concentrations at 100 pM and 1 nM, with recoveries of 95.32% and 101% and coefficients of variation of 3.9% and 4.1%. These results suggest that possible interferences from matrix on fusion gene detection are negligible assuming to the fact the complex components in serum did not interfere with the assembly of nanotweezers and subsequent activation of MNAzyme for catalytic cleavage. Thus, the assay system has high potential for detecting fusion gene in complex biological samples.

## 4. Conclusions

In summary, a nonenzymatic fluorescent method has been fabricated for sensitive and specific detection of fusion gene based on a novel functional nanotweezer. Various molecular events correspond to different signal inputs of the “AND” logic gate, which determine the structure state of the engineered tweeter and further activity of DNAzyme. These concessive responses result in a signal switching effect, attaining a logic gate operation. In this strategy, complicated molecular events occurred in the cell can be readably interpreted with the different logic outputs based on the engineered nanodevice. The sensitivity and applicability of the nanosystem were tested with the BCR/ABL fusion fragment as a target model, and results demonstrated its high potential for fusion gene measurement with a detection limit of 5.29 pM. Furthermore, to realize real biological detection, we first need to get DNA or RNA transcript followed by incubating the generated transcript with the nanotweezer system for signal conversion and amplified detection. Therefore, the smart nanosystem is expected to have high potential in analyzing fusion gene.

## Figures and Tables

**Table 1:** Interference investigation via spiking BCR/ABL fusion gene into 100-fold diluted healthy human serum with six parallel experiments.

Spiked	Found	Recoveries	Coefficients of variation
50 pM	48.16 pM	96.32%	5.0%
100 pM	95.32 pM	95.32%	3.9%
1 nM	1.01 nM	101.00%	4.1%
5 nM	4.92 nM	98.40%	3.6%

## Data Availability

The data used to support the findings of this study are available from the corresponding author upon request.

## Supplementary Materials

Supplementary Materials Table S1: oligonucleotides used in the present work.
