# Supplementary material for: DNA Nanotweezers with Hydrolytic Activity for Enzyme-Free and Sensitive Detection of Fusion Gene via Logic Operation
Source: J Anal Methods Chem. 2018 Oct 18;2018:4178045. doi: 10.1155/2018/4178045 (PMC6211150; doi:10.1155/2018/4178045)
Supplement: Supplementary Materials — Table S1: oligonucleotides used in the present work. [file 4178045.f1.doc]

**DNA nanotweezers with hydrolytic activity for enzyme-free and sensitive detection of fusion gene via logic operation**

Yongjie Xu^a, 1,^ *, Xiangrong Luo^a, 1^, Nana Geng^b^, Mingsong Wu^b^, Zhishun Lu^a,^ *

*^a^ Department of Laboratory Medicine, Guizhou Provincial People's Hospital;College of Basic Medicine, Guizhou University,Guiyang 550002, Guizhou, China*

*^b^ Special Key Laboratory of Oral DiseasesResearch, Higher Education Institutions of Guizhou Province; Zunyi Medical University, Zunyi  563099, Guizhou, China*

Table S1Oligonucleotides used in the present work

| Oligonucleotides | Sequences (5 '-3 ') |
| --- | --- |
| BCR/ABL | CAGATGCTACTGGCCGCTGAAGGGCTTTTGAACTCTGCTTAAATCCAGTGGCTGAGTGG |
| BCR | AACCCTCCTCCCCAAACCAGTACTTACTTGAACTCTGCTTAAATCCAGTGGCTGAGTGG |
| ABL | CAGATGCTACTGGCCGCTGAAGGGCTTCTGGAAGAGAAAGGGGGGAACAGAAAAAAGAA |
| Random | GACACCAGAAGCAGCAACAACGATTGTTTCGCCAATGAAGACATATTCTTCTGCGCCAG |
| CA-3 (strand A) | ***ACCTT*GGTCGCTCTTACAAGGC**AACAACGAGAGGAAAC |
| CA-4 | ***TACCTT*GGTCGCTCTTACAAGGC**AACAACGAGAGGAAAC |
| CA-5 | ***GTACCTT*GGTCGCTCTTACAAGGC**AACAACGAGAGGAAAC |
| CA-6 | ***AGTACCTT*GGTCGCTCTTACAAGGC**AACAACGAGAGGAAAC |
| CA-7 | ***CAGTACCTT*GGTCGCTCTTACAAGGC**AACAACGAGAGGAAAC |
| CB-3 (strand B) | CCAGGGAGGCTAGCT**ATCCGAAGCATTCCAGGT*TTGGT*** |
| CB-4 | CCAGGGAGGCTAGCT**ATCCGAAGCATTCCAGGT*TTGGTA*** |
| CB-5 | CCAGGGAGGCTAGCT**ATCCGAAGCATTCCAGGT*TTGGTAC*** |
| CB-6 | CCAGGGAGGCTAGCT**ATCCGAAGCATTCCAGGT*TTGGTACT*** |
| CB-7 | CCAGGGAGGCTAGCT**ATCCGAAGCATTCCAGGT*TTGGTACTG*** |
| CC (strand C) | CACTGGATTTAAGCAGAGTTCAA**TGCCTTGTAAGAGCGACCATCAACCTGGAATGCTTCGGAT**AAGCCCTTCAGCGGCCAGTAG |
| Fluorescence substrates (strand F) | Cy5-GTTTCCTCguCCCTGG-BHQ1 |

The portion in orange is complementary sequences on the hinge region. The underline portion is binding bases for MNAzyme and fluorescence substrates.
